# Outcome of Autologous Matrix Induced Chondrogenesis (AMIC) in cartilage knee surgery: data of the AMIC Registry

**DOI:** 10.1007/s00402-012-1621-5

**Published:** 2012-10-16

**Authors:** J. Gille, P. Behrens, P. Volpi, L. de Girolamo, E. Reiss, W. Zoch, S. Anders

**Affiliations:** 1 Department of Trauma and Reconstructive Surgery, University Of Schleswig-Holstein, Campus Lübeck, Lübeck, Germany; 2 CUNO Hamburg, Hamburg, Germany; 3 Istituto Clinico Humanitas, Milan, Italy; 4 IRCCS Istituto Ortopedico Galeazzi, Milan, Italy; 5 OrthoPraxis Oftringen, Oftringen, Switzerland; 6 Heidekreis Klinikum, Soltau, Germany; 7 Rheuma- und Orthopädiezentrum, Bad Abbach, Germany; 8 Department of Trauma and Orthopaedics, University of Schleswig-Holstein, Ratzeburger Allee 160, 23538 Lübeck, Germany

**Keywords:** AMIC, Cartilage, Knee, Surgery, Lysholm score

## Abstract

**Introduction:**

Autologous Matrix-Induced Chondrogenesis (AMIC) is an innovative treatment for localized full-thickness cartilage defects combining the well-known microfracturing with collagen I/III scaffold. The purpose of this analysis was to evaluate the medium-term results of this enhanced microfracture technique for the treatment of chondral lesions of the knee.

**Methods and materials:**

Patients treated with AMIC (Chondro-Gide^®^, Geistlich Pharma, Switzerland) were followed using the AMIC Registry, an internet-based tool to longitudinally track changes in function and symptoms by the Lysholm score and VAS.

**Results:**

A series of 57 patients was enrolled. The average age of patients (19 females, 38 males) was 37.3 years (range 17–61 years). The mean defect size of the chondral lesions was 3.4 cm^2^ (range 1.0–12.0 cm^2^). All defects were classified as grade III (*n* = 20) or IV (*n* = 37) according to the Outerbridge classification. Defects were localized at the medial (*n* = 32) or lateral (*n* = 6) condyle, at the trochlea (*n* = 4) and at the patella (*n* = 15). The follow-up period was 2 years. The majority of patients were satisfied with the postoperative outcome, reporting a significant decrease of pain (mean VAS preop = 7.0; 1 year postop = 2.7; 2 years postop = 2.0). Significant improvement of the mean Lysholm score was observed as early as 1 year after AMIC and further increased values were noted up to 2 years postoperatively (preop. 50.1, 1 year postop. 79.9, 2 year postop. 85.2).

**Conclusions:**

AMIC is an effective and safe method of treating symptomatic chondral defects of the knee. However, further studies with long-term follow-up are needed to determine if the grafted area will maintain structural and functional integrity over time.

**Level of evidence:**

Prognostic study, Level IV.

## Introduction

The limited healing potential of articular cartilage is a well-known problem in orthopedic surgery [26]. Cartilage degeneration may be accompanied by pain, immobility, stiffness, loss of quality of life and can potentially lead to severe osteoarthritis in the long term. A variety of surgical techniques that aim for resurfacing and regenerating of articular cartilage have evolved.

Increasing the intrinsic repair has traditionally been focused on the recruitment of chondrogenic cells (MSCs) from the bone marrow by penetration of the subchondral bone by drilling or microfracturing (MFx) [20]. Currently, microfracturing is the most commonly used cartilage repair procedure in cartilage defects [2]. But the deficiencies of fibrocartilaginous repair tissue inevitably lead to breakdown under normal joint loading [32]. In microfracturing, chondrogenic cells (MSCs) migrate in the fibrin network of the blood clot [8]. However, the fibrin clot is not mechanically stable to withstand the tangential forces [11]. An implanted exogenous scaffold (e.g. a collagen matrix) may improve the mechanical stability and durability for endogenous cells and may provide a proper stimulus for chondrogenic differentiation and cartilage regeneration. Autologous Matrix-Induced Chondrogenesis (AMIC^®^) combines microfracturing with a collagen I/III matrix (Chondro-Gide^®^, Geistlich Pharma AG, Wolhusen, Switzerland). The AMIC procedure provides two major advantages; on one hand, it is a one-step procedure with no need of cartilage harvesting potentially leading to donor site morbidity and on the other, it is cost effective with no need of in vitro cell expansion [4]. In a recent clinical trial we were able to prove that AMIC is an effective and safe method for treating symptomatic chondral defects of the knee [16].

The aim of the present study is to update our experience with the AMIC technique based on the data of the AMIC registry.

## Materials and methods

Study subjects consisted of a cohort of patients treated with AMIC (Chondro-Gide^®^, Geistlich Pharma, Switzerland) and enrolled in the AMIC registry since 2005. The registry is a multicenter program designed to longitudinally track changes in function and symptoms by the Lysholm score and VAS. Documentation is done on electronic forms and surgeons have access via a Web interface. The communication protocol is SSL encrypted. Surgeons have only access to their own patients’ data and summary and overall performance data is anonymized and de-identified. Patient participation in the registry is voluntary. All participants were informed and consented for recording and storing of their data. There is no radiographic follow-up after AMIC in the registry. Consequently, data regarding the development of radiographically verified osteoarthritis are not available. Radiographic follow-ups were not done because of financial constraints and also to avoid placing demands on the surgeons that go beyond their own follow-up routines. More advanced investigations (e.g. gait analysis and muscle strength) are also omitted for the same reasons.

Patients were included in the analysis if they had an AMIC-treated (Outerbridge grade III or IV) lesions in the knee; data were collected at baseline and at specified follow-up times. The main exclusion criteria from the analysis were underlying rheumatic disease, total meniscectomy and revision surgery after the index procedure.

The operative procedure was performed through a mini-open approach as described previously [16].

Baseline data collection included surgical history, defect origin, size and location of lesions, concurrent procedures, age, weight, and sex. Therapeutic success was assessed on the basis of two different scores: at baseline and follow-up, patients rated their pain using the Visual Analog Scale (VAS), with 0 indicating “no pain” and 10 indicating “pain as bad as it could possibly be”. The Lysholm score is a well-validated functional score and was chosen for follow-up [16]. Investigators and research assistants made extensive efforts to locate subjects, motivate them to stick to the follow-up protocol and mail follow-up questionnaires to patients. Patients were not financially compensated for their time and effort to complete data collection forms.

### Statistical analysis

The two primary outcomes (pain and Lysholm score) are described by showing means and SD at each time point of assessment (baseline, 1 and 2 years post-operative). Furthermore, their mean improvements (along with SD) between these three time points were also calculated. For each of the two scores and each time interval (baseline to 1 year, 1–2 years and baseline to 2 years), we used the two-sided Wilcoxon signed rank tests to test the corresponding null hypotheses of no systematic improvement. These six tests were performed at the level of 5 %/6 = 0.833 % each to bound the global type 1 error probability to 5 % (Bonferroni correction for multiple testing).

Exploratory testing at the 5 % level of hypotheses related to the two primary outcomes were carried out without correction for multiple testing: two-sided Mann–Whitney *U* test and, where applicable, Kruskal–Wallis test were used for testing the null hypotheses of no systematic differences between subgroups (sex, age etc.) in improvement from baseline to 2 years. In addition, Wilcoxon signed rank tests were performed to test the null hypothesis of no systematic improvements between baseline and 2 years within subgroup.

## Results

Fifty-seven patients with complete data sets at 2-year follow-up could be extracted from the Registry and included in the present analysis. Patient data are as follows: 19 females, 38 males; mean BW: female 60 kg (range 40–110) and male 85.5 kg (range 63–118); mean age: 37.3 years (range 17–61 years).

The mean defect size of the chondral lesions was 3.4 cm^2^ (range 1–9 cm^2^). All defects were classified as grade III (*n* = 20) or IV (*n* = 37) according to the Outerbridge classification [27]. The defects were situated on the medial femoral condyle (*n* = 32), on the lateral femoral condyle (*n* = 6), on the patella (*n* = 15) and at the trochlea (*n* = 4). The chondral lesions were of traumatic origin in 16 patients (28 %) and idiopathic in 41 patients (72 %). In 35 patients (61 %) the right knee and in 22 patients (39 %) the left knee was treated. Previous surgical procedures (*n* = 35) were diagnostic arthroscopies (*n* = 10), partial meniscectomies (*n* = 5), shaving (*n* = 16) and drilling or microfracture (*n* = 4). When performing the AMIC procedure, concomitant surgical procedures such as a patella realignment surgery (*n* = 2), corrective osteotomies (*n* = 3), partial meniscectomies (*n* = 6) and anterior cruciate ligament reconstruction (*n* = 1) were performed. No complications or adverse events occurred in the cases studied.

**Fig. 1:**
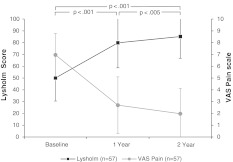
Mean and standard deviation values of the clinical outcome evaluated by the Lysholm and VAS score. Both scores improved significantly from baseline at 2 years post-operative (Lysholm 35.1 ± 19.6, *p* < 0.001; VAS 4.7 ± 2.7, *p* < 0.001)

There was significant improvement of knee pain from the mean baseline value of 7.0 ± 1.8 (range 1 –10) to the score at the 1-year follow-up (mean 2.7 ± 2.4, range 0–9, *p* < 0.001) and at the 2-year follow-up (mean 2.0 ± 2.1, range 0–9, *p* = 0.003). The mean VAS improvement from baseline to 1 year was 4.2 ± 2.6 (*p* < 0.001), from 1 to 2 years 0.5 ± 2.3 (*p* = 0.003), and from baseline to 2 years 4.7 ± 2.7 (*p* < 0.001). Results of the VAS are summarized in Fig. 1.

The mean preoperative Lysholm score was 50.1 ± 19.6 (range 9–79). A significant improvement was seen in the follow-up at 1 year with a mean 79.9 ± 21.2 (range 17–100, *p* < 0.001) and at 2 years with a mean 85.2 ± 18.4 (range 27–100, *p* = 0.002). The mean improvement from baseline to 1 year was 24.2 ± 31.7 (*p* < 0.001), from 1 to 2 years 11.0 ± 26.1 (*p* = 0.002), and from baseline to 2 years 35.1 ± 19.6 (*p* < 0.001). Figure 1 summarizes the results of the Lysholm score.

**Fig. 2:**
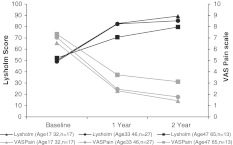
Effect of age on post-operative clinical outcome evaluated by Lysholm and VAS score. Patients were divided in three subgroups as depicted. Mean scores at baseline and at 1 and 2-year follow-up for the three subgroups are indicated. No statistically significant between-group differences were observed for Lysholm (*p* = 0.085) and VAS (*p* = 0.338)

In order to determine the influence of age on the postoperative results, patients were divided into three subgroups: patients between 17 and 32 years (group A, *n* = 17), patients between 33 and 46 years (group B, *n* = 27) and patients between 47 and 65 years (group C, *n* = 13). Results are shown in Fig. 2. Mean Lysholm score improvement from baseline to 2 years was significant in all groups (group A: 39.3 ± 20.3, *p* < 0.001; group B: 36.2 ± 21.2, *p* < 0.001; group C: 27.5 ± 11.9, *p* = 0.001). No statistically significant between-group differences from baseline to 2 years were observed (*p* = 0.085) with younger patients showing better results. The mean VAS score also improved significantly in all groups at 2 years after surgery (group A: 5.1 ± 2.0, *p* < 0.001; group B: 5.0 ± 2.6, *p* < 0.001; group C: 3.7 ± 3.2, *p* = 0.003). Between-group comparison showed better pain improvement, although not significant, in younger patients compared to their older counterparts (*p* = 0.338).

To elucidate the impact of body weight on the clinical outcome, patient subgroups were formed as follows for analysis: males with a body weight of more (*n* = 18) or less (*n* = 12) than 90 kg and females with a body weight of more (*n* = 5) or less (*n* = 14) than 70 kg. Body weight was not found to significantly influence the improvement of the Lysholm score (males 34.2 ± 19.0 and 34.2 ± 12.3, *p* = 0.485; females 42.2 ± 15.6 and 34.9 ± 27.2, *p* = 0.517) or the VAS (males 4.5 ± 2.3 and 6.0 ± 2.4, *p* = 0.068; females 3.8 ± 2.2 and 4.0 ± 2.4, *p* = 0.816) 2 years after the index procedure.

In order to investigate the impact of the defect size on the clinical outcome, patients’ were divided into 3 subgroups: group A: defect size >0–3 cm^2^, group B: defect size >3–6 cm^2^, group C: defect size >6–9 cm^2^. The between-group results did not differ significantly either in Lysholm (*p* = 0.703) or VAS (*p* = 0.969) scores. The mean outcome improvement measured by the Lysholm and VAS score 2 years after the index procedure was 34.8 ± 21.1 and 5.0 ± 1.9 in group A, 33.6 ± 17.1 and 4.4 ± 3.4 in group B and 36.4 ± 19.3 and 4.8 ± 2.3 in group C.

**Fig. 3:**
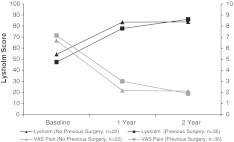
Mean Lysholm and VAS score in patients without and with previous surgery. Mean improvement from baseline in both scores was comparable in the two groups (Lysholm 29.4 ± 19.6 and 38.7 ± 18.8, *p* = 0.276; VAS 4.6 ± 2.3 and 4.9 ± 2.9, *p* = 0.465)

In order to investigate whether the score results were dependent on the number of previous operations, the patients were divided into 2 subgroups (no previous operation (*n* = 22) and previous operations (*n* = 35). There were no significant differences in Lysholm score improvement from baseline to 2 years (no previous operation: 29.4 ± 19.4; previous operation: 38.7 ± 19.1, *p* = 0.276) and in VAS pain reduction (no previous operation: 4.6 ± 2.3; previous operation: 4.9 ± 2.8, *p* = 0.465). Results are shown in Fig. 3. Previous surgery affecting the subchondral lamina (e.g. drilling, microfracture) did not significantly influence the outcome (*n* = 10; mean Lysholm score preop: 42.0 ± 22.9; 1-year follow-up: 81.8 ± 22.6; 2-year follow-up: 86.0 ± 18.6). The mean VAS score improvement in this group at the 2-year follow-up was 5.8 ± 2.5.

**Fig. 4:**
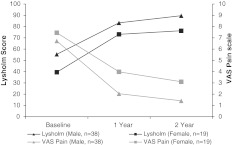
Effect of sex on clinical outcome evaluated by the Lysholm and VAS score. Mean scores at baseline and at 1 and 2-year follow-up are shown. Between-group differences of mean improvement from baseline for Lysholm (female: 36.8 ± 25.9; male: 34.3 ± 15.7, *p* = 0.416) and VAS (female: 4.0 ± 2.3; male: 5.1 ± 2.8, *p* = 0.047) were not significant

There were no significant differences between male and female patients in Lysholm score improvement from baseline to 2 years (female: 36.8 ± 25.9; male: 34.3 ± 15.7, *p* = 0.416) and in VAS pain reduction (female: 4.0 ± 2.3; male: 5.1 ± 2.8, *p* = 0.047). For both sexes a significant improvement of Lysholm score values and a significant decline of the VAS values were seen at follow-up. Figure 4 illustrates the sex-specific differences of the Lysholm score and the VAS.

## Discussion

The AMIC (Autologous Matrix-Induced Chondrogenesis) technique was first described in 2003 by Behrens et al. and at present it is widely used on the one hand in its original form and on the other hand with further developments [2, 5]. Although AMIC is a well-established treatment in cartilage defect therapy, only sparse published evidence is found in the literature [16, 24, 31]. In a follow-up trial on 27 patients treated with AMIC in its original form, a significant improvement of all clinical scores was seen and 87 % of the patients were highly satisfied with the results after surgery [16]. MRI analysis showed moderate-to-complete filling with a normal-to-incidentally hyperintense signal in most cases [16]. A recent prospective study with 17 patients and a 36-month follow-up reports an improvement of the Lysholm score from preoperative 38 to 74 at follow-up [31]. 76.5 % of the patients were satisfied or extremely satisfied with their functional results [31].

To update our experience with the AMIC technique, the present analysis evaluates mid-term results based on the AMIC Registry, which is an internet database to longitudinally track changes in function and symptoms by the Lysholm score and VAS. For selection of patients from the Registry for analysis in the present study, we used the criteria of availability of complete patient data and post-operative results at 2 years. A group of 57 patients met these criteria and their data were analysed.

The key finding in the present analysis is that an enhanced microfracture technique (AMIC) is well suited for the treatment of patients with focal cartilage defects. Mean Lysholm score values were 85 points at 2-year follow-up compared with mean preoperative values of 50 points. Patients reported significant pain improvement (VAS) from baseline to 2-year follow-up with a mean 4.7 points. This shows that the loading capacity of the treated knee joint is well maintained at midterm. The results are in accordance with our prior published data about AMIC in cartilage defect therapy of the knee with a follow-up time up to 60 months; thus, the required 24 months to obtain the final regenerate quality is fulfilled in this series [6]. The status of the patients 2 years after cartilage repair is considered an important indicator for the future outcome [23].

Well-established rating systems have been used in this analysis to summarize relevant outcome measures. The combination of the Lysholm score and the VAS have been recommended in the literature before [14]. The Lysholm scoring system has demonstrated validity, reliability and responsiveness to cartilage pathology and treatment [14]. The VAS has widely been used to monitor subjective satisfaction postoperatively [13]. The KOOS score (Knee Injury and Osteoarthritis Outcome Score) was added to the AMIC Registry in 2009, so that only short-term follow-up data are currently available using this scoring system and thus these data were not analysed the suitability of the KOOS score for assessing post-operative clinical outcome in cartilage repair procedures has been demonstrated [3].

Our data demonstrates that, there are patient-specific and defect-specific factors that influence clinical outcomes after AMIC. According to available literature, younger patients with no concomitant ligamentous instability, meniscal deficiency or patellofemoral malalignment, can expect the best outcome [19].

Independent of the patients′ age, the Lysholm score improved in this study, but younger patients showed better results. This is in accordance with the literature, rating age as a significant predictor of outcome following cartilage repair [19]. For example, after matrix-induced autologous chondrocyte implantation (MACI), significantly better modified Cincinnati knee scores were seen at the time of the 1-year follow-up in those who were less than 35 years old compared to their older counterparts [1].

In our series, no significant impact of the cartilage defect size on the outcome measures was seen. In a previous series, patients with a defect size more than 8 cm^2^ did not benefit from the enhanced microfracture procedure [16]. Although our data do not support this conclusion, we believe that the AMIC technique has limited success rates in extensive cartilage defects, as described e.g. for autologous chondrocyte implantation (ACI) [22]. A size-based clinical outcome association was shown when ACI was compared with microfracture. At 2 years after microfracture, patients with defects measuring <4 cm^2^ had significantly better Lysholm scores and VAS scores than patients with larger lesions [22].

In a former series, cartilage repair was more efficacious in males compared to their female counterparts [16]. This fact is not supported by the current data. Little is known about sex-specific differences in cartilage repair. Further studies should elucidate this aspect for a better understanding of sex-related dimorphism in knee pathology and improvement of related surgical treatments. In other fields, a gender specific research is already on its way [7, 30].

Our results do not support published literature showing better outcome in patients with fewer previous surgical procedures prior to cartilage repair. Surgical procedures prior to cartilage repair were reported to have a significant impact on postoperative clinical outcome [1], but not in the context of AMIC [16]. It needs to be emphasized that in our series previous surgery affecting the subchondral lamina (e.g. drilling, microfracture) did not negatively influence the outcome, as reported previously [1]. To our knowledge, none of the patients had to be revised within the 2-year follow-up. Besides this, concurrent surgery to address meniscal lesions did not negatively affect the clinical outcome of AMIC [19].

Although microfracture demonstrated significantly better improvement as compared with e.g. autologous chondrocyte implantation at short-term follow-up, the beneficial results were not maintained at longer follow-up despite equivalent clinical outcomes, indicating deterioration with time after microfracture [21]. A recent systematic review of 28 clinical studies on the clinical outcomes after microfracture confirmed that microfracture peaks early and deteriorates with time [25]. Currently no prospective, randomized trial is available to compare microfracture and AMIC. A potential superiority of the AMIC technique compared to microfracture especially in the long-term follow-up would demonstrate the positive effect of the collagen-I/III-matrix. These advantages are at the moment only based on experimental data [15, 17].

Current studies suggest modifications to the original AMIC technique and a switch from conventional microfracturing awls to drilling with *K* wires [28] [18]. Distinct differences between microfracture and drilling for acute subchondral bone structure and osteocyte necrosis were seen and additional ongoing studies suggest these differences may significantly affect long-term cartilage repair outcomes [9]. Most AMIC procedures are performed with a mini open approach, but an all-arthroscopic AMIC procedure of cartilage defects in the knee has been described [29]. A modified AMIC technique (called AMIC plus technique) was described by Dhollander et al. [10]. The combination of the AMIC technique with platelet-rich plasma gel resulted in a clinical improvement in all patients, but was not demonstrated by MRI findings [10]. In summary, modifications of the original AMIC technique may improve cartilage repair outcome and optimize the operative approach, but long-term and randomized studies are mandatory to confirm the initial results and the reliability of modified AMIC techniques.

There are limitations that need to be acknowledged and addressed regarding the present analysis. The first limitation concerns the heterogeneous patient population, which reflects the situation of patients with an indication for cartilage repair surgery. But from another point of view, these data are representative of the general cartilage patient population. In contrast, randomized controlled trials do not necessarily match the majority of patients [12]. The second limitation has to do with the extent to which the findings can be generalized beyond the cases studied. The number of cases is very limited for broad generalization. In general, the AMIC Registry provides detailed data, but it is also important to emphasize what the AMIC Registry is not able to show. There is no radiographic follow-up and consequently the development of osteoarthritis cannot be monitored. An important limitation in registries is a bias due to gaps in follow-up; our experience has shown that it is difficult to motivate both patients who are completely satisfied with restitutio ad integrum of the knee as well as those with persistent pain and malfunction of the knee to keep the follow-up appointments. However, these limitations can also be seen as fruitful avenues for future research.

## Conclusion

We present here the mid-term results after AMIC in a selected set of patients from the AMIC Registry. The key finding is that AMIC is feasible for the treatment of cartilage defects in the knee. The cases analyzed showed a gradual and significant clinical improvement at follow-ups 1 and 2 years after surgery.
